# Characterization of a novel microRNA, miR-188, elevated in serum of muscular dystrophy dog model

**DOI:** 10.1371/journal.pone.0211597

**Published:** 2019-01-30

**Authors:** Hiroyuki Shibasaki, Michihiro Imamura, Sayuri Arima, Jun Tanihata, Mutsuki Kuraoka, Yasunari Matsuzaka, Fumiaki Uchiumi, Sei-ichi Tanuma, Shin’ichi Takeda

**Affiliations:** 1 Department of Molecular Therapy, National Institute of Neuroscience, National Center of Neurology and Psychiatry, Kodaira, Tokyo, Japan; 2 Department of Gene Regulation, Faculty of Pharmaceutical Sciences, Tokyo University of Science, Noda, Chiba, Japan; 3 Department of Cell Physiology, The Jikei University School of Medicine, Minato, Tokyo, Japan; 4 Laboratory of Experimental Animal Science, Nippon Veterinary and Life Science University, Musashino, Tokyo, Japan; 5 Department of Medical Molecular Informatics, Meiji Pharmaceutical University, Kiyose, Tokyo, Japan; 6 Department of Genomic Medicinal Science, Research Institute for Science and Technology, Organization for Research Advancement, Tokyo University of Science, Noda, Chiba, Japan; University of Minnesota Medical Center, UNITED STATES

## Abstract

MicroRNAs (miRNAs) are non-coding small RNAs that regulate gene expression at the post-transcriptional level. Several miRNAs are exclusively expressed in skeletal muscle and participate in the regulation of muscle differentiation by interacting with myogenic factors. These miRNAs can be found at high levels in the serum of patients and animal models for Duchenne muscular dystrophy, which is expected to be useful as biomarkers for their clinical conditions. By miRNA microarray analysis, we identified miR-188 as a novel miRNA that is elevated in the serum of the muscular dystrophy dog model, CXMD_J_. miR-188 was not muscle-specific miRNA, but its expression was up-regulated in skeletal muscles associated with muscle regeneration induced by cardiotoxin-injection in normal dogs and mice. Manipulation of miR-188 expression using antisense oligo and mimic oligo RNAs alters the mRNA expression of the myogenic regulatory factors, MRF4 and MEF2C. Our results suggest that miR-188 is a new player that participates in the gene regulation process of muscle differentiation and that it may serve as a serum biomarker reflecting skeletal muscle regeneration.

## Introduction

MicroRNAs (miRNAs) are evolutionary conserved small non-coding RNAs composed of approximately 22 nucleotides, and that function in the post-transcriptional regulation of gene expression. Specific interaction of miRNAs with complementary sequences at 3’ noncoding regions of messenger RNAs (mRNAs) causes mRNA degradation or inhibition of protein translation, resulting in negative regulation of gene expression. [1, 2] The miRNA database, miRBase (http://www.mirbase.org/), has recently listed more than 35,000 miRNAs from a variety of species. In mammals, miRNAs are predicted to regulate about 60% of genes [3], which means that various biological phenomena are relevant to miRNA expression and comprehensive studies of miRNA function are thus important.

Skeletal muscle development occurs through characteristic cell processes, i.e., proliferation and migration of progenitor cells, differentiation of the cells into myoblasts, formation of myotubes by fusion of arrested myoblasts, and maturation of myotubes into myofibers [4]. These processes are predominantly regulated by several myogenic regulatory factors (MRFs) that belong to the basic helix-loop-helix (bHLH) family of transcriptional factor (MyoD, Myf5, myogenin, and MRF4) together with other transcriptional factors, i.e., Pax3, Pax7, and MEF2 family proteins [5]. Recent studies have shown that various miRNAs can play roles in crucial processes of skeletal myogenesis. Muscle-specific miRNAs, i.e., miR-1, miR-133, and miR-206 have been characterized as myogenic regulators [6].

Interestingly, we and other groups previously reported that these three miRNAs are strongly expressed in the serum of Duchenne muscular dystrophy (DMD) and its animal models, suggesting that they could act as novel biomarkers for muscular dystrophy [7–10]. Some non-muscle-specific miRNAs are also known to be myogenic regulators and are elevated in the serum of muscular dystrophy [9, 11, 12]. These observations suggest that search for serum miRNAs related to myogenesis may lead to establishing of markers reflecting the pathological condition of skeletal muscle in muscle disorders.

In the present study, we performed miRNA microarray assay using the serum of a dystrophic dog model for DMD and found a novel miRNA, miR-188, which was elevated from around the onset stage up to several months. Our analyses did not identify miR-188 as a muscle-specific-type of miRNA, but revealed that it originated from immature muscle cells at the early stage of muscle regeneration. In vitro analysis using the C2C12 myogenic cell line indicated a correlation between miR-188 elevation and expression of myogenic transcription factors, suggesting that miR-188 is a novel regulator of muscle differentiation.

## Materials and methods

### Animals

All animals used in this study were housed in facilities of the National Institute of Neuroscience, National Center of Neurology and Psychiatry. All dogs were born into the canine X-linked muscular dystrophy in Japan (CXMD_J_) colony at the General Animal Research Facility, National Institute of Neuroscience, National Center of Neurology and Psychiatry [13]. Dogs were treated in accordance with the guidelines provided by the Ethics Committee for the Treatment of Middle-sized Laboratory Animals (Approval ID: 28–02, 29–02). Mice were supplied by Clea Japan Inc. (Tokyo, Japan). Mice were treated in accordance with the guidelines provided by the Ethics Committee for the Treatment of Laboratory Animals (Approval ID: 2016001).

### Antibodies and reagents

Rabbit polyclonal antibody against MEF2-interacting transcription repressor, MITR (ab59718), and laminin (L9393) were purchased from Abcam (Cambridge, UK) and Sigma-Aldrich (St. Louis, MO), respectively. Mouse monoclonal antibodies against ubiquitin-conjugating enzyme E2I, UBE2I (610748), Glyceraldehyde-3-phosphate dehydrogenase, GAPDH (sc-32233), and developmental myosin heavy chain, dMyHC (NCL-MHCd), were purchased from BD Bioscience (San Jose, CA), Santa Cruz Biotechnology (Santa Cruz, CA), and Leica Biosystems (Newcastle, UK), respectively. Affinity purified peroxidase-conjugated anti-mouse IgG goat antibody (PNIM0817) and peroxidase-conjugated anti-rabbit IgG goat antibody (A0545) were purchased from Merck (Darmstadt, Germany) and Beckman Coulter Diagnostics (Fullerton, CA), respectively. Alexa488-conjugated anti-mouse IgG goat antibody (A-11029) and Alexa568-conjugated anti-rabbit IgG goat antibody (A-11036) were purchased from Thermo Fisher Scientific (Waltham, MA). Gel Red dye for nucleus labelling was purchased from Biotium Inc. (Haywood, CA). Cardiotoxin (CTX) was purchased from Sigma-Aldrich. Synthesized single-strand anti-miR188 (MH12963) and miRNA inhibitor negative control (4464076) oligo RNAs, and double-strand mimic miR188 (MC12963) and miRNA mimic negative control (4464058) oligo RNAs were purchased from Ambion (Austin, TX).

### Serum and tissues collection

Serum samples were prepared from 14 dogs (7 normal and 7 dystrophic dogs), when those dogs were aged 3 weeks, 2 months, 6 months, 9 months, and 1 year.

Tissue collection was carried out after euthanizing the dog as described previously [14]. Dog tissues, including diaphragm, left ventricle, hippocampus, kidney, lung, liver, spleen, intestine, and colon were collected from 3 normal and 3 dystrophic dogs at 2 months of age. Tibialis cranialis (TC) muscles were collected from 3 normal and 3 dystrophic dogs at the ages of 0 days, 3 weeks, 2 and 6 months, and 1 year, respectively. All tissue samples were frozen in 2-methylbutane chilled by liquid nitrogen and stored at -80°C until usage. In the analysis of CTX-injury dog muscles, we used the frozen stocked samples those were previously reported [14].

Sixteen 8-week-old mice were used for induction of muscle regeneration by CTX-injection. Mice were anaesthetized by isoflurane inhalation, and 100 μl of CTX solution (10 μM CTX in phosphate-buffered saline) was injected into the tibialis anterior (TA) muscle of 12 mice. As a control, 100 μl of phosphate-buffered saline (PBS) was injected into the TA muscles of 4 mice. TA muscles were isolated from four mice each at 3, 5, and 7 days after CTX-injection.

### Cell culture and transfection

Mouse muscle C2C12 cells were maintained as undifferentiated myoblasts in growth medium (GM: DMEM supplemented with 10% fetal calf serum) at 37°C in an atmosphere of 5% CO_2_ and 95% humidity. Cells were plated onto 6-well plates, and when they had proliferated to about 60% confluence, cells were transfected with synthesized anti-sense miR-188, mimic miR-188, or their control RNAs (Ambion) using Lipofectamine RNAiMAX reagent (Thermo Fisher Scientific) according to the manufacturer’s instructions. At 24 hours after the transfection, growth medium was switched to differentiation medium (DMEM supplemented with 2% horse serum, 100 U/ml penicillin, and 100 μg/ml streptomycin). Three days later, cells were washed with Tris-buffered saline (TBS) and then treated with Trizol reagent (Invitrogen, Carlsbad, CA) for preparation of total RNAs. For the purpose of immunoblot analysis, cells were treated with heated lysis solution (2% sodium dodecyl sulfate [SDS], 125 mM Tris-HCl, pH 6.8, 15% glycerol) and protein concentration was determined using a protein assay (Bio-Rad Laboratories Inc., Hercules, CA), as described previously [15].

### Quantification of fusion index

C2C12 myotubes on 6-well plates were fixed in PBS containing 4% formaldehyde and 0.1% Triton X-100 at room temperature for 10 min. The cells were washed with TBS and incubated with anti-MyHC (MF-20) antibody (1:40 dilution in TBS containing 2% casein) overnight at 4°C, followed by incubation with Alexa488-conjugated goat anti-mouse secondary antibody (1:600 dilution) and Gel Red Dye (1: 10,000 dilution) for visualization of the nuclei at room temperature for 30 min. The stained cells were examined with an inverted fluorescence microscope (IX50; Olympus Ltd., Tokyo, Japan). The percentage of nuclei in MHC-positive myotubes with more than 2 nuclei was calculated. Each datum under different culture condition was obtained from elected microscopic 3 fields containing in total at least 2000 nuclei.

### miRNAs microarray analysis

miRNA microarray analysis was performed using total RNAs prepared from dystrophic and normal dog serums at 2 months of age. To avoid detection of individual differences in miRNA expression, serum samples of 3 dystrophic dogs and 3 normal dogs were mixed, respectively, and used for total RNA preparation using 3D-Gene RNA extraction reagent (Toray Industries Inc., Tokyo, Japan) according to the manufacturer’s instructions. Extracted total RNAs were labeled with fluorescence using 3D-Gene miRNA labeling kit (Toray Industries Inc.), and which were hybridized onto 3D-Gene 4 animal miRNA Oligo chips mounted with 291 dog miRNAs (Toray Industries Inc.). Obtained fluorescent signals on the chip were analyzed using 3D-Gene Extraction software (Toray Industries Inc.). The microarray data are in agreement with the Minimum Information About a Microarray Experiment (MIAME) and are publicly available through the Gene Expression Omnibus (GEO) database (http://www.ncbi.nlm.nih.gov/projects/geo/) under the accession number GSE123567.

### Reverse transcription-quantitative PCR

Total RNA was extracted from dog sera using miRNeasy Mini Kit (Qiagen, Hataworth, CA), and from dog tissues, mouse muscles, and C2C12 cultured cells using Trizol reagent according to the manufacturer’s instructions. In order to measure expression levels of 18S rRNA and mRNAs, total RNA was reverse transcribed into cDNA using PrimeScript RT reagent Kit (Takara, Tokyo, Japan). Quantification PCR for the cDNA was performed using TB Green Premix Ex Taq II (Takara) in accordance with the manufacturer’s instructions. Primer sequences for 18S rRNA and mRNAs are listed in S2 Table. cDNA of small nucleolar RNAs (snoRNAs) and mRNAs was produced using miScript RT kit (Qiagen). The synthesized cDNA was quantified using miScript SYBR Green Kit (Qiagen) in accordance with the manufacturer’s instructions. miScript primer assays (Qiagen) were used for specific primers of snoRNAs and miRNAs (S3 Table). The PCR and monitoring of SYBR green signals were performed using by CFX Connect system (Bio-Rad). Relative expression levels of mRNAs, miRNAs and snoRNAs were calculated using the 2^-ΔΔCt^ method [16]. Expression levels of 18S rRNA, sno202, and sno234 were used as internal controls and for normalization of expression of dog and mouse mRNAs, dog miRNA, and mouse miRNA, respectively.

### Immunoblot analysis

Preparation of C2C12 cell lysates for immunoblot analysis was as described above. SDS-polyacrylamide gel electrophoresis (SDS-PAGE) and protein transfer to a polyvinylidene difluoride (PVDF) membrane were performed as described previously [15]. The protein-transferred membrane was incubated with anti-MITR antibody (1:200 dilution in TBS containing 2% casein) or anti-UBE2I antibody (1:200 dilution). Immunoreactive protein bands were visualized using the chemiluminescence detection system for standard-type Amersham ECL (GE Healthcare UK, Ltd). Signals of MITR and UBE2I were quantified by Image J software (http://imagej.nih.gov/ij/), and were normalized with expression levels of GAPDH.

### Histology

Cryosections of dog TC muscles were subjected to haematoxylin and eosin (HE) staining as described previously [14].

Immunohistochemistry was performed using cryosections (8 μm) of dog TC muscles. The cryosections were fixed in cold acetone and equilibrated with TBS at room temperature. The sections were double-stained with anti-dMyHC mouse antibody (1:40 dilution in TBS containing 2% casein) and anti-laminin rabbit antibody (1:30 dilution) overnight at 4°C, followed by visualization with Alexa488-conjugated goat anti-mouse secondary antibody (1:600 dilution) and Alexa568-conjugated goat anti rabbit secondary antibody (1:1000 dilution) at room temperature for 30 min. Fluorescence signals on the muscle sections were observed under the inverted fluorescence microscope (Olympus IX50). To quantify the number of dMyHC-positive muscle fibers in each age dog, the percentage of dMyHC and laminin double-positive fibers in total laminin-positive fibers was calculated. At least 1000 muscle fibers were counted on multiple randomly selected microscopic fields at each specimen.

### Statistical analysis

Difference between groups were analyzed by Student’s t-test, Holm multiple test, and Dunnett’s test using BellCurve for Excel (SSRI Ltd., Tokyo, Japan). *P*-values < 0.05 were considered to be statistically significant.

## Results

### Search for microRNAs showing unique expression in serum of dog model of Duchenne muscular dystrophy

**Fig 1:**
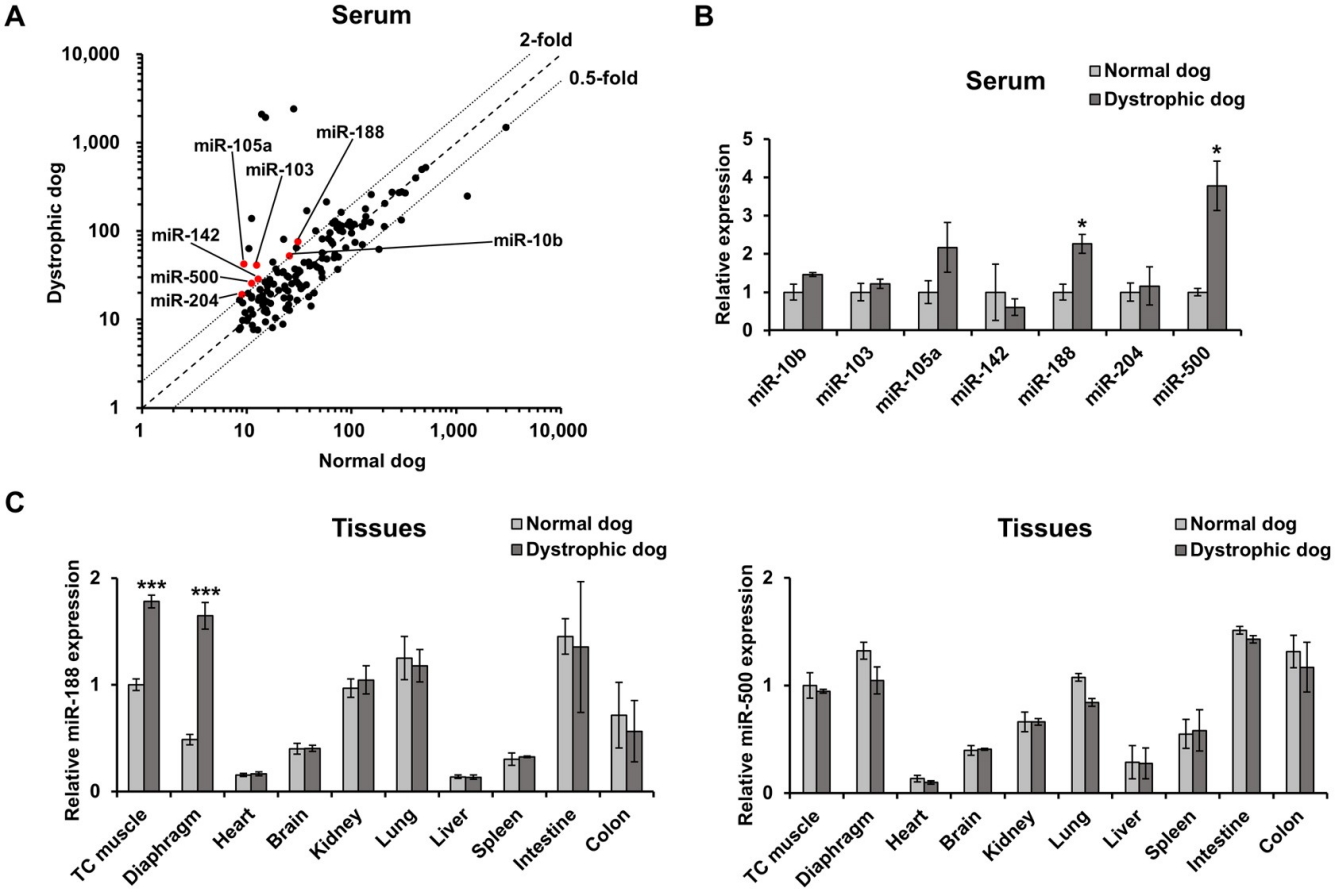
Search for miRNAs that are elevated in serum and skeletal muscles of muscular dystrophy. (A) Scatterplot of microarray data comparing relative levels of miRNAs in serum from dystrophic dogs (CXMD_J_) and normal dogs at the age of onset (2 months). Newly detected 7 miRNAs are indicated in red. (B) RT-qPCR validation of 7 elevated microRNAs in muscular dystrophy dog model. miRNAs from 100 μl of dystrophic and normal dog serum were reverse transcribed into cDNA, and which was subjected to expression analysis by RT-qPCR (n = 3). (C) Expression analysis of miR-188 and miR-500 in the tissues of normal and dystrophic dogs at the age of 2 months by RT-qPCR (n = 3). Data represent mean ± standard error (SE). Statistical analysis was performed using Student’s t-test; *P < 0.05, ***P < 0.001 compared to normal dogs.

In order to identify serum miRNAs that show specific expression in DMD, we performed the miRNA microarray analysis using serums from dystrophic dogs at 2 months of age, when starts to display the onset of clinical symptoms [17] (Fig 1A and S1 Table). One hundred and forty-two miRNAs were detected by the microarray in both normal and dystrophic sera. Among these, 19 miRNAs were elevated by more than 2-fold in dystrophic sera when compared to normal serum, while 8 miRNAs were reduced by half or more. Seven of the elevated miRNAs have not been reported previously as serum biomarkers of DMD and the animal models. Validation of this result by reverse transcription-quantitative PCR (RT-qPCR) confirmed a significant difference in the elevation of 2 miRNAs, i.e., miR-188 and -500 (Fig 1B). Expression analysis of these 2 miRNAs using dog tissues clearly showed up-regulation of miR-188 expression in dystrophic skeletal muscles but not in other tissues (heart, brain, kidney, lung, liver, spleen, intestine, and colon), when compared to normal (Fig 1C). Whereas, in all tissues examined, no significant differences were observed in the expression of miR-500 between dystrophic and normal control dogs. These findings suggest that the specific elevation of miR-188 in the serum reflects abnormalities in dystrophic skeletal muscles. We then focused on miR-188 and investigated the relationship between its expression and the pathological condition of skeletal muscles.

### Elevation of miR-188 expression during muscle regeneration

**Fig 2:**
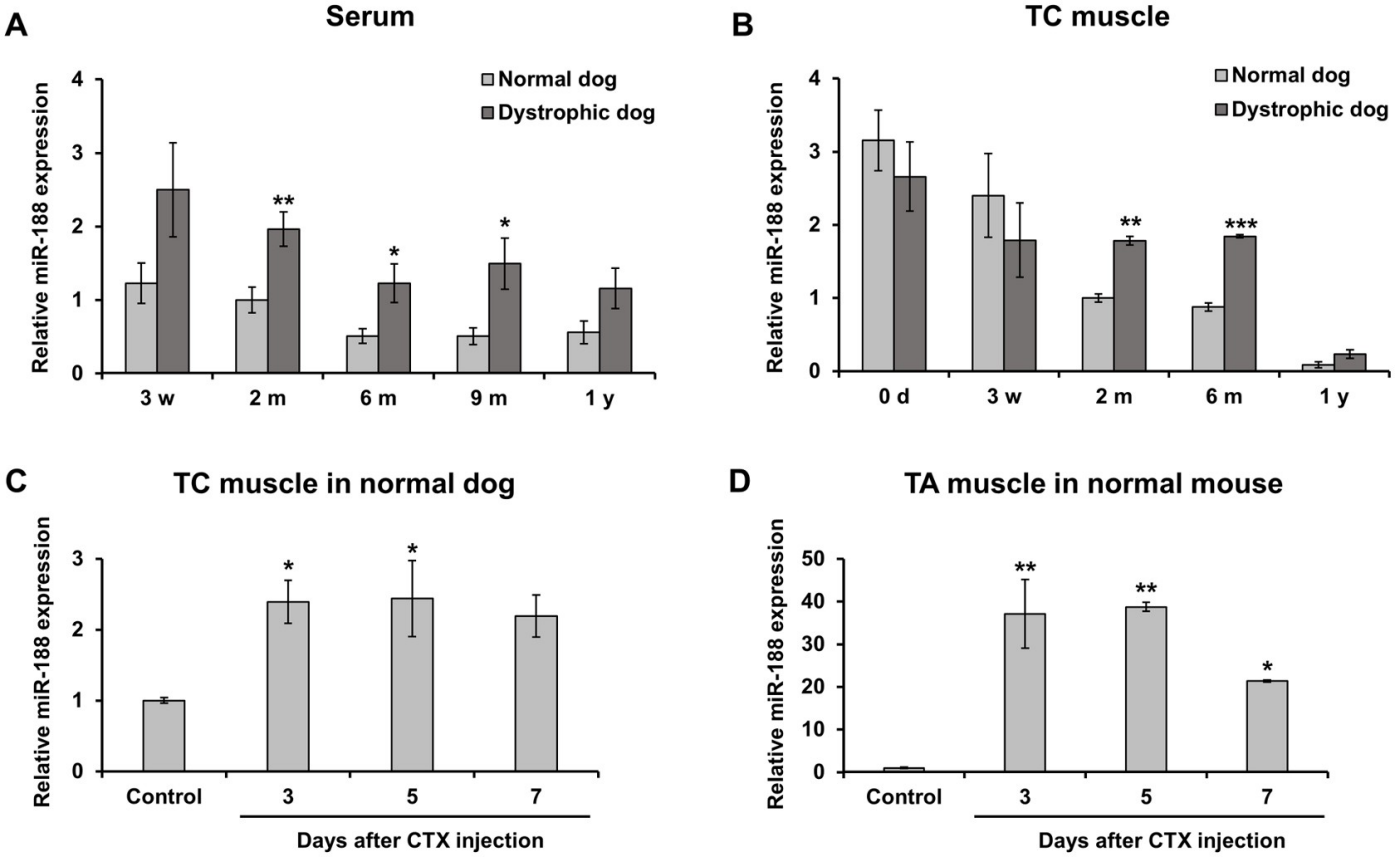
Changes in miR-188 expression during muscle regeneration. (A) Expression patterns of miR-188 in serum from normal and dystrophic dogs at 3 weeks (w), 2 months (m), 6 months, 9 months, and 1 year (y) of age were analyzed by RT-qPCR (n = 7 each). (B) Expression patterns of miR-188 in TC muscles of normal and dystrophic dogs at 0 days (d), 3 w, 2 m, 6 m, and 1 y of age were analyzed by RT-qPCR (n = 3). (C) miR-188 expression in CTX-injured TC muscles in normal dogs was measured by RT-qPCR (n = 3). (D) miR-188 expression of CTX-injury TA muscles of normal mice was measured by RT-qPCR. Data represent mean ± SE. Statistical analysis was performed using Student’s t-test with the Holm multiple test for A and B, and Dunnett’s test for C and D; *P < 0.05, **P < 0.01, ***P < 0.001 compared to normal dogs or controls.

**Fig 3:**
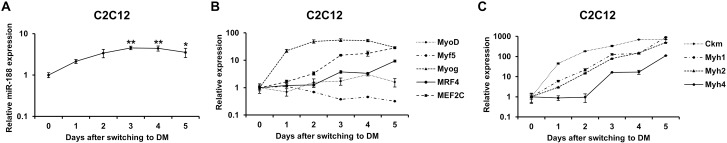
Expression pattern of miR-188 in C2C12 myoblasts during muscle differentiation. (A) Changes in miR-188 expression in differentiating C2C12 cells after switching to differentiation medium (DM) were analyzed by RT-qPCR (n = 4). (B) Expression patterns of 5 myogenic transcriptional factors, i.e., MyoD, Myf5, myogenin (Myog), MRF4, and MEF2C during C2C12 cell differentiation (n = 3). (C) Expression patterns of four differentiation markers, Ckm, Myh1, 2, and 4 during C2C12 differentiation. Data represent means ± SE. Statistical analysis was performed using Dunnett’s test; *P < 0.05, **P < 0.01 compared to 0 day.

In order to reveal the expression pattern of miR-188 associated with progression of muscular dystrophy, we examined its expression in serums and TC muscles during the growth of dystrophic and normal dogs by RT-qPCR (Fig 2A and 2B). The analysis showed that miR-188 expression in dystrophic dogs is higher than in normal dog serum at the ages of 2, 6, and 9 months, but not at 3 weeks and 1 year of age, which agrees with the results of miR-188 quantitative analysis with dog TC muscles. We checked pathology of the dog TC muscles and found marked abnormalities in 2- and 6-month-old dystrophic dogs (S1 Fig). These findings strongly suggest that the significant elevation of serum miR-188 in dystrophic dogs is derived from lesions in skeletal muscles, i.e., muscle degeneration, regeneration, and infiltration of monocytes and so on. Interestingly, even in normal dogs, remarkable miR-188 expression was observed in newborn muscles, and expression levels was gradually decreased with the growth of dogs. Immunostaining of developmental myosin heavy chain (dMyHC), which is an immature and regeneration muscle fiber marker, on both of dystrophic and normal dog TC muscles showed the proportions of dMyHC-positive fibers were very high in newborn and 3-week-old dogs (S2A and S2B Fig). In dystrophic dog TC muscles at the ages of 2 and 6 months and 1 year, dMyHC-positive fibers were frequently observed, and which were estimated at 33%, 51%, and 18% of total muscle fibers, respectively. The changes of dMyHC-positive fibers were similar to the expression pattern of miR-188 in TC muscles (Fig 2B), suggesting that miR-188 is up-regulated in immature muscle cells during muscle regeneration. To test this, we examined the pattern of miR-188 expression during injury and regeneration of normal dog muscle by CTX-injection (Fig 2C). CTX injury induced extensive myofiber necrosis and infiltration of inflammatory cells, including neutrophils, within 1 day. Mononuclear myogenic cells largely replaced neutrophils within 3 days and regenerated myofibers were readily identified at around day 5. qPCR analysis indicated miR-188 up-regulation at days 3 and 5 after CTX-injection. Almost the same results were also obtained by CTX-injury analysis in TA muscles of normal mice (Fig 2D). These results strongly suggest that miR-188 is up-regulated at early stages of muscle differentiation. Therefore, we examined miR-188 expression during muscle differentiation *in vitro* using the C2C12 mouse myogenic cell line (Fig 3A). After setting the cells in culture medium for differentiation, miR-188 expression gradually increased with time and showed significant up-regulation from day 3 to day 5. Comparative study with known muscle differentiation regulators and differentiation markers revealed that the expression pattern of miR-188 was similar to the patterns of myogenic regulatory factor 4 (MRF4), myocyte enhancer factor 2C (MEF2C), and myosin heavy chain isoform 1, 2, and 4 (Myh1, 2, and 4) (Fig 3B and 3C).

### Effects of miR-188 on expression of myogenic factors during C2C12 myoblast differentiation

**Fig 4:**
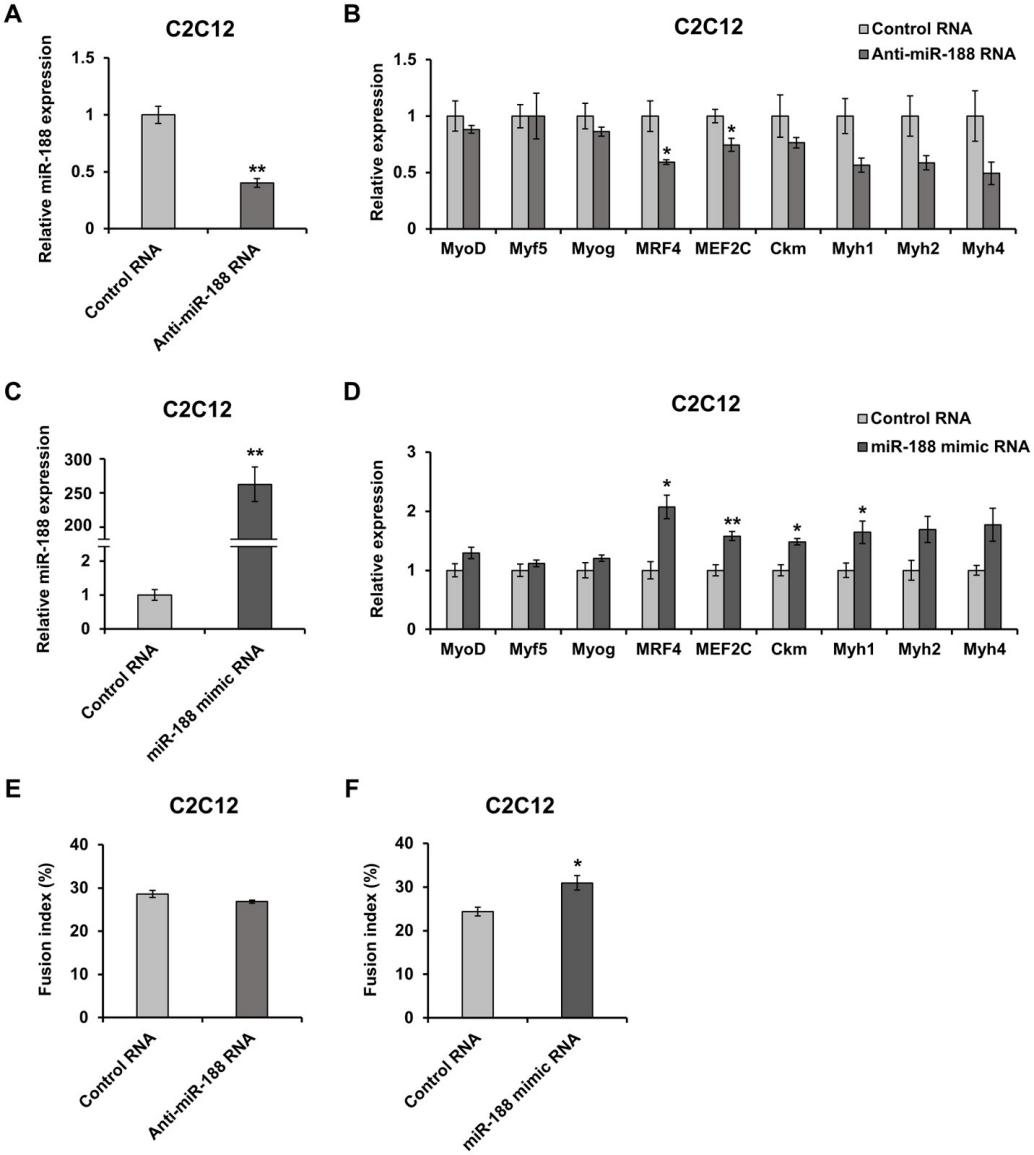
Effects of miR-188 on mRNA expression of myogenic factors during C2C12 differentiation. (A) Inhibitory effects of anti-miR-188 oligo RNA on expression of endogenous miR-188 in C2C12 cells. After 4 days of culture following transfection with anti-miR-188 RNA (3-day culture in DM), relative expression levels of endogenous miR-188 were analyzed by RT-qPCR (n = 3). Control RNA has a sequence designed not to target any human, mouse, and rat genes. (B) Effects of anti-miR-188 oligo RNA on expression of 5 myogenic transcriptional factors and 4 muscle differentiation markers in C2C12 cells after 3-day culture in DM. Relative expression levels of mRNAs were analyzed by RT-qPCR (n = 3). (C) Measurement of total miR-188 in C2C12 cells after 4-day culture following transfection with mimic miR-188 oligo RNA (3-day culture in DM). The amounts of total miR-188 RNA in the C2C12 cells were measured by RT-qPCR (n = 3). (D) Effects of mimic miR-188 oligo RNA on expression of myogenic transcriptional factors and muscle differentiation markers were examined as described in B. (E, F) Effects of antisense and mimic oligo RNAs on C2C12 fusion index under the same experimental conditions as shown in B and D (n = 3). Data represent mean ± SE. Statistical analysis was performed using Student’s t-test; *P < 0.05, **P < 0.01 compared to control.

We further examined whether the elevation of miR-188 alters the expression of myogenic transcriptional factors and differentiation markers during C2C12 myoblast differentiation. Transfection of synthesized antisense oligo RNA inhibited miR-188 expression by more than 50% after 3 days of culture in differentiation medium, while mimic miR-188 introduced into the cells was estimated at about 250-fold when compared to endogenous miR-188 (Fig 4A and 4C). The inhibitory treatment of C2C12 significantly decreased expression of MRF4 and MEF2C, but not other MRFs (MyoD, Myf5, and myogenin) (Fig 4B). Whereas, the treatment tended to suppress expression of Myh1, 2, and 4. In the analysis of myotube formation, the miR-188 inhibition did not show the significant inhibitory effect on fusion index (Fig 4E). In overexpression experiments with miR-188 mimic, MRF4, MEF2C, muscle creatine kinase (Ckm), and Myh1 were significantly up-regulated, but Myh2 and Myh4 tended to increase (Fig 4D). On the other hand, the mimic miR-188 expression significantly increased fusion index (Fig 4F). As myogenic transcriptional factors and myosin heavy chain transcripts have no target sequences for miR-188, these results suggest that miR-188 indirectly regulates the expression through other molecules.

### Search for molecules that directly interact with miR-188 during muscle differentiation

**Fig 5:**
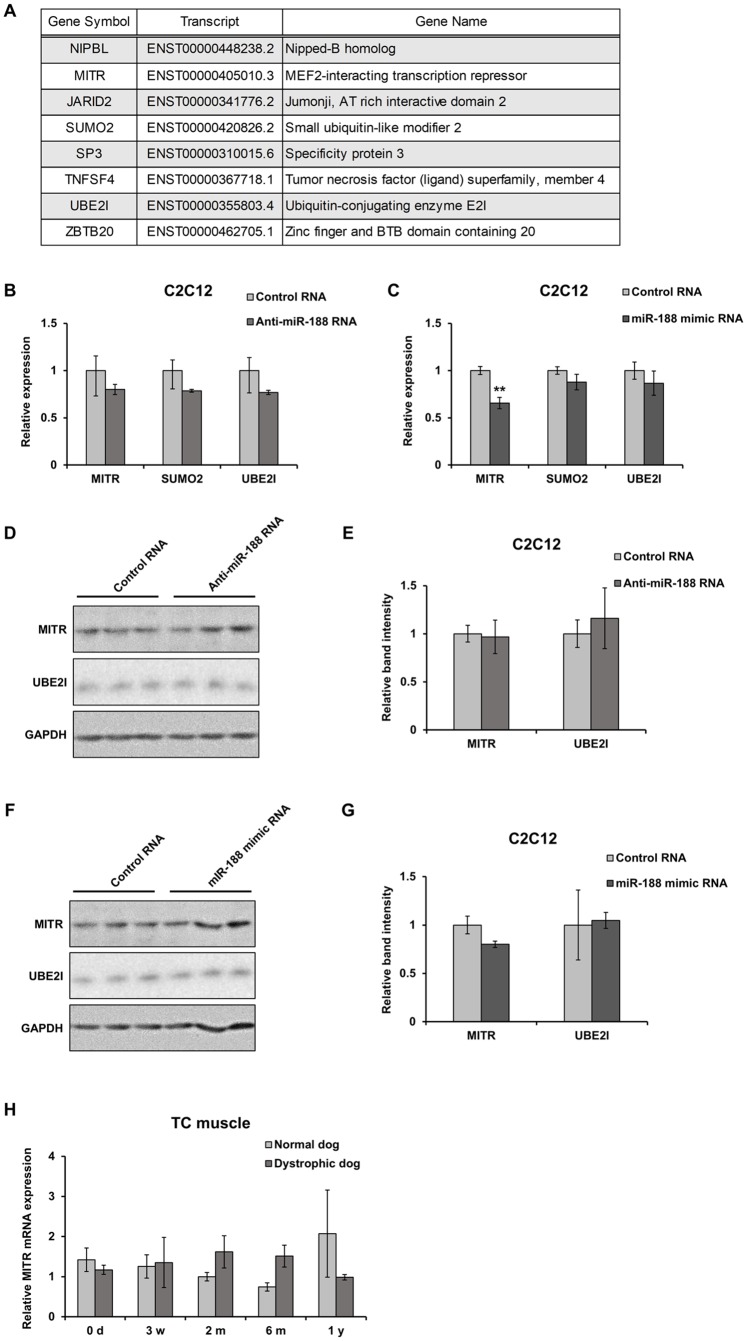
Protein expression of candidates for miR-188 target in C2C12 cells. (A) List of miR-188 target molecules containing predicted sequences to bind to miR-188 at 3’noncoding region. (B) Effects of anti-miR-188 oligo RNA on expression of MITR, SUMO2, and UBE2I mRNAs in C2C12 cells after 3-day culture in DM. Relative expression levels of mRNAs were analyzed by RT-qPCR (n = 3). (C) Effects of mimic miR-188 oligo RNA on mRNA expression were examined as described in B (n = 3). (D) Expression levels of MITR and UBE2I proteins in C2C12 cells under the same culture conditions as in B. Protein signals were detected by immunoblotting. (E) Signal intensities of MITR and UBE2I in D were quantified using Image J software. Relative amounts were calculated by normalizing with the expression levels of GAPDH. (F) Expression levels of MITR and UBE2I proteins in C2C12 cells under the same culture conditions as that of C. (G) Signal intensities of MITR and UBE2I in F were quantified by the same method as described in E. (H) Expression patterns of MITR mRNA in TC muscles of normal and dystrophic dogs at 0 days (d), 3 weeks (w), 2 months (m), 6 months, and 1 year (y) of age were analyzed by RT-qPCR (n = 3). Data represent mean ± SE. Statistical analysis was performed using Student’s t-test; **P < 0.01 compared to controls.

In order to identify target molecules of miR-188, we initially search for candidates having miR-188-binding sequences using TargetScan 7.1 (http://www.targetscan.org/vert_71/). The software program listed 141 candidate molecules. We then attempted to narrow down the candidates based on the condition that it negatively regulates transcription by gene ontology analysis using the Database for Annotation, Visualization and Integrated Discovery (DAVID: https://david.ncifcrf.gov/), which revealed 8 transcripts: NIPBL, MITR, JARID2, SUMO2, SP3, TMFSF4, UBE2I, and ZBTB20 (Fig 5A). Among these, MITR, SUMO2, and UBE2I have been reported to be involved in transcription of myogenic factors, and we therefore focused on these molecules. Inhibition of miR-188 did not change expression levels of these 3 mRNAs when compared to the control experiment (Fig 5B). However, overexpression of miR-188 significantly reduced mRNA expression levels of MITR, but not SUMO2 and UBE2I (Fig 5C). Furthermore, we examined the expression levels of protein products of the genes. Immunoblot analysis did not clearly show the changes in protein levels of these candidate molecules (Fig 5D and 5E), but overexpression of miR-188 tended to reduce MITR protein levels (Fig 5F and 5G). To further examine the possibility that MITR expression is negatively regulated by miR-188, we quantified MITR expression in TC muscles during the growth of dystrophic and normal dogs. MITR expression did not change in transcriptional levels (Fig 5H).

## Discussion

In this study, we found that miR-188 was specifically elevated in the serum of CXMD_J_ dogs with onset of muscular dystrophy. Significant elevation of serum miR-188 was observed until around 9 months of age, and which was compatible with the results of expression analysis using skeletal muscle. The period of miR-188 elevation with CXMD_J_ growth was corresponding to the time when the muscle regeneration was vigorous. Even in normal animals, induction of muscle regeneration by CTX-injury indicated significant miR-188 elevation. Taken together with the fact that miR-188 was up-regulated in C2C12 cells during differentiation, our results revealed that miR-188 elevation in CXMD_J_ serum reflects muscle regeneration, implying that miR-188 is useful as a marker for such regeneration.

Microarray and RT-qPCR analyses also indicated significant elevation of miR-500 in the CXMD_J_ serum. Changes in serum levels showed the same pattern as for miR-188 (S3 Fig and Fig 2A), suggesting a relationship between miR-500 and muscle regeneration. Thus, miR-500 may be a better biomarker for muscle regeneration than miR-188, because the elevation of miR-500 in CXMD_J_ serum was much higher than that of miR-188. However, the origin of miR-500 and the mechanism of its elevation remain unclear. Thus, further analysis is needed.

*In vitro* analysis using C2C12 cells showed that overexpression of miR-188 increased fusion index (Fig 4F). The result suggested that miR-188 may participate in the myogenic differentiation of the cells, but suppression of miR-188 expression did not show an effect on the fusion index. On the other hand, the mRNA expression of MRF4 and MEF2C were clearly affected by both treatment of the mimic and antisense oligo RNAs in C2C12 cells, as shown in Fig 4B and 4D, suggesting that miR-188 would contribute to myotube maturation rather than myotube formation. Indeed, the expression of mRNA encoding mature MyHC, such as Myh1, Myh2, and Myh4 has been changed by the treatment of them.

MRF4 expression is known to be regulated by MEF2 family members (MEF2A, MEF2C, and MEF2D) [18, 19], whereas MEF2C expression is also reported to be regulated by these members, including MEF2C itself [20]. Simultaneous knockout of MEF2 family genes (*Mef2a*, *Mef2c*, and *Mef2d*) clearly showed down-regulation of MRF4 expression, but also reduced the expression of Ckm, Myh1, and Myh4 [21], and it was similar to changes in MRF4 and muscle differentiation marker molecules in the expression analysis using C2C12 cells (Fig 4B and 4D).

Since there were no target sequences for miR-188 in the 3’-untranslated regions of MRF4 and MEF2C and miRNA is a negative regulator of gene expression, regulatory effect of miR-188 on these factors is likely to be mediated through other molecule(s). We listed 141 molecules that have target sequences for miR-188 in their transcripts using TargetScan, and narrowed our search down to three candidates (MITR, UBE2I, SUMO2) based on the condition that they negatively regulate myogenic transcriptional factors in muscle differentiation, as miRNA is a negative regulator of gene expression. miR-188 was already proved to regulate the MITR protein levels by directly binding to 3’ non-coding region of its mRNA [22]. During C2C12 differentiation, overexpression of miR-188 significantly reduced mRNA expression of MITR, but the alteration of the MITR protein levels were quite limited (Fig 5C, 5F and 5G), while inhibition of miR-188 did not change the expression levels of MITR mRNA and protein (Fig 5B, 5D and 5E). *In vivo* analysis, MITR expression levels did not change in TC muscles between normal and dystrophic dogs and during the growth. These results were not convincing to prove that MITR was the direct target of miR-188 in skeletal muscle cell, although protein levels of MITR in dog muscles were not clear.

In this study, UBE2I and SUMO2 were also expected to be direct targets of miR-188 during muscle differentiation. However, UBE2I mRNA and protein levels did not change as a result of miR-188 manipulation. SUMO2 could not be verified, because the protein could not be detected by the anti-SUMO2 antibody we used. Sumoylation of MEF2 by SUMO2 has been reported to reduce MEF2 activity [23]. Therefore, miR-188 may inhibit sumoylation of MEF2 to maintain MEF2 activity by suppressing translation of SUMO2. PTEN could be one of other candidate targets, which inhibits PI3K/Akt signaling related to MEF2 [24, 25], therefore should be examined in the future.

There has been, however, still the possibility that MITR may be involved as a target of miR-188 in skeletal muscle. During murine muscle regeneration induced by CTX injection, miR-188 expression was markedly increased (Fig 2D), compared with the modest increase of miR-188 in C2C12 differentiation (Fig 3A). These results suggested that the increase of miR-188 expression during muscle regeneration may be derived not only from expression in myogenic cells but also from that in mesenchymal progenitors and/or macrophages. In bone marrow mesenchymal stem cells (BMSCs), MITR has been reported to be a direct target of miR-188 [22]. Based on this finding, it is possible that MITR may be involved in other cells such as mesenchymal progenitors in skeletal muscles. Further analysis with another experimental system is necessary to prove this hypothesis.

In conclusion, we report that miR-188 is elevated in the serum of muscular dystrophy model dogs and is up-regulated in response to muscle regeneration. In vitro analysis using myoblasts shows that miR-188 positively regulates myogenic transcriptional factors and muscle differentiation markers. These findings suggest that miR-188 is a novel regulator of muscle differentiation and has potential as a marker for regeneration.

## Supporting information

S1 Fig Histopathology of dog TC muscles. TC muscle sections of normal and dystrophic dogs at 0 days, 3 weeks, 2 months, 6 months, and 1 year of age were subjected to HE staining. Scale bar: 100 μm.
(TIF)

S2 Fig Expression of dMyHC in dog TC muscles. (A) Immunofluorescence double staining of (green) and laminin (red) in cryosections from normal and dystrophic dog TC muscles at 0 days, 3 weeks, 2 months, 6 months, and 1 year of age. Scale bar: 100 μm. (B) The percentages of dMyHC-positive fibers in TC muscles on the same time course as A were calculated (n = 3). Data represent mean ± SE. Statistical analysis was performed using Student’s t-test with the Holm multiple test; *P < 0.05, **P < 0.01, ***P < 0.001 compared to normal dogs.
(TIF)

S3 Fig Changes in miR-500 expression in dog serum accompanying growth. Expression patterns of miR-500 in serum of normal and dystrophic dogs at ages of 3 weeks, 2 months, 6 months, 9 months, and 1 year were analyzed by RT-qPCR (n = 7 each). Data represent means ± SE. Statistical analysis was performed using Student’s t-test with the Holm multiple test; *P < 0.05, ***P < 0.001 compared to normal dogs.
(TIF)

S1 Table miRNA microarray data. List of signal intensities globally normalized on serum miRNA microarray. ND indicates that miRNA was not detected.
(PDF)

S2 Table List of primer sets for 18S rRNA and mRNAs. (PDF)

S3 Table List of primer sets for snoRNAs and miRNAs. (PDF)
