# Supplementary figures and images for: Characterization of a novel microRNA, miR-188, elevated in serum of muscular dystrophy dog model
Source: PLoS One. 2019 Jan 30;14(1):e0211597. doi: 10.1371/journal.pone.0211597 (PMC6353185; doi:10.1371/journal.pone.0211597)

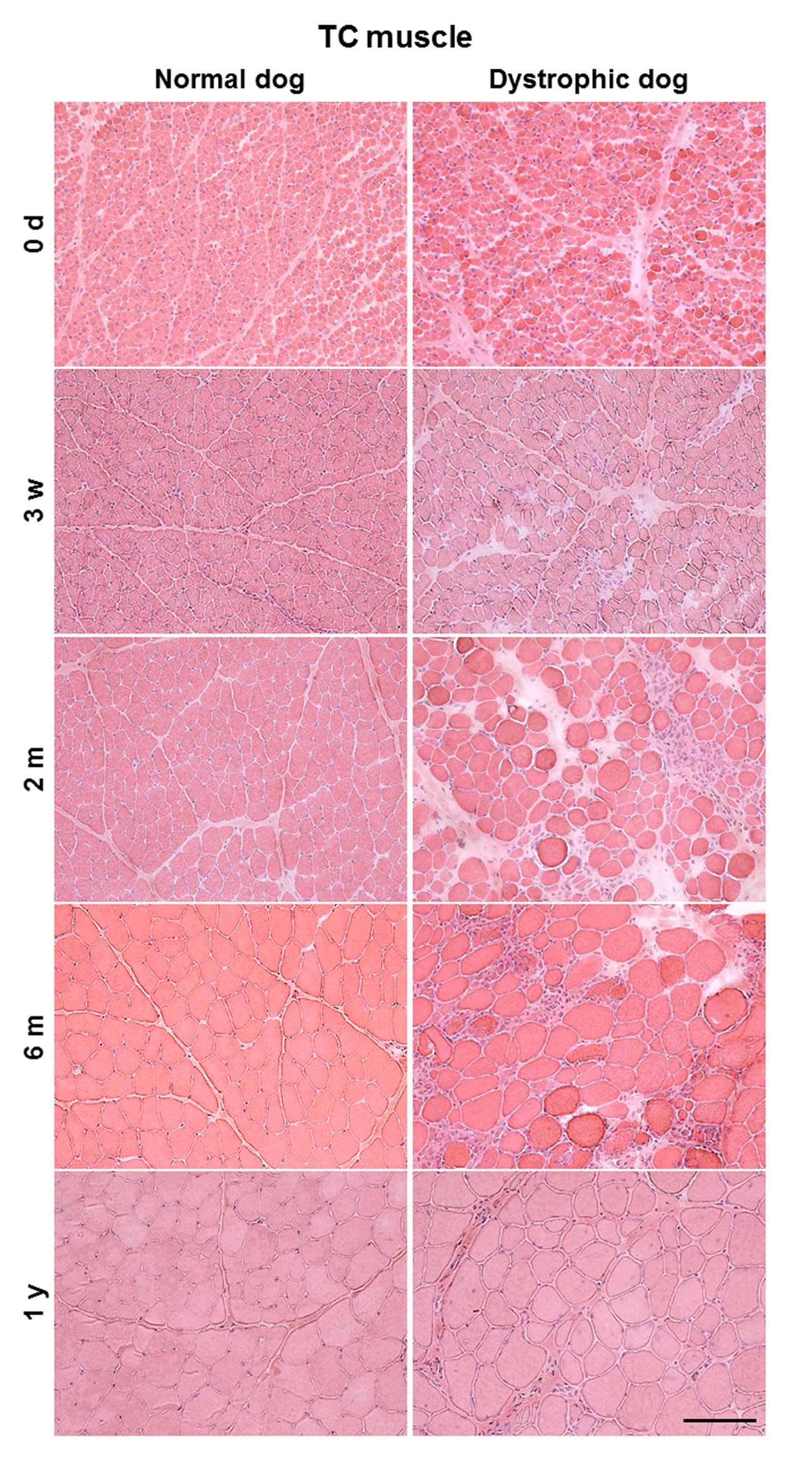

Supplement: S1 Fig — TC muscle sections of normal and dystrophic dogs at 0 days, 3 weeks, 2 months, 6 months, and 1 year of age were subjected to HE staining. Scale bar: 100 μm. (TIF) [file pone.0211597.s001.tif]

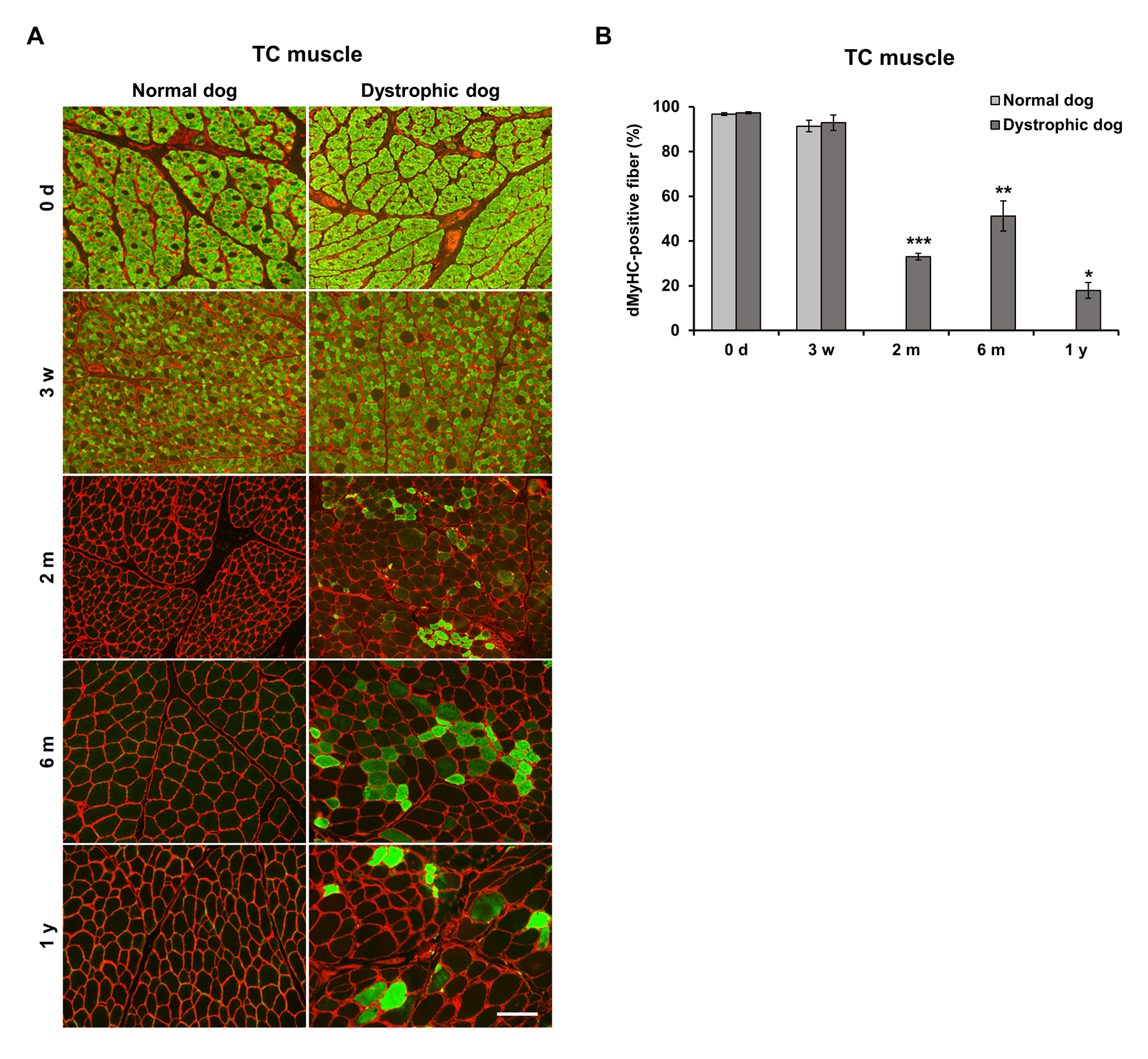

Supplement: S2 Fig — (A) Immunofluorescence double staining of (green) and laminin (red) in cryosections from normal and dystrophic dog TC muscles at 0 days, 3 weeks, 2 months, 6 months, and 1 year of age. Scale bar: 100 μm. (B) The percentages of dMyHC-positive fibers in TC muscles on the same time course as A were calculated (n = 3). Data represent mean ± SE. Statistical analysis was performed using Student’s t-test with the Holm multiple test; *P < 0.05, **P < 0.01, ***P < 0.001 compared to normal dogs. (TIF) [file pone.0211597.s002.tif]

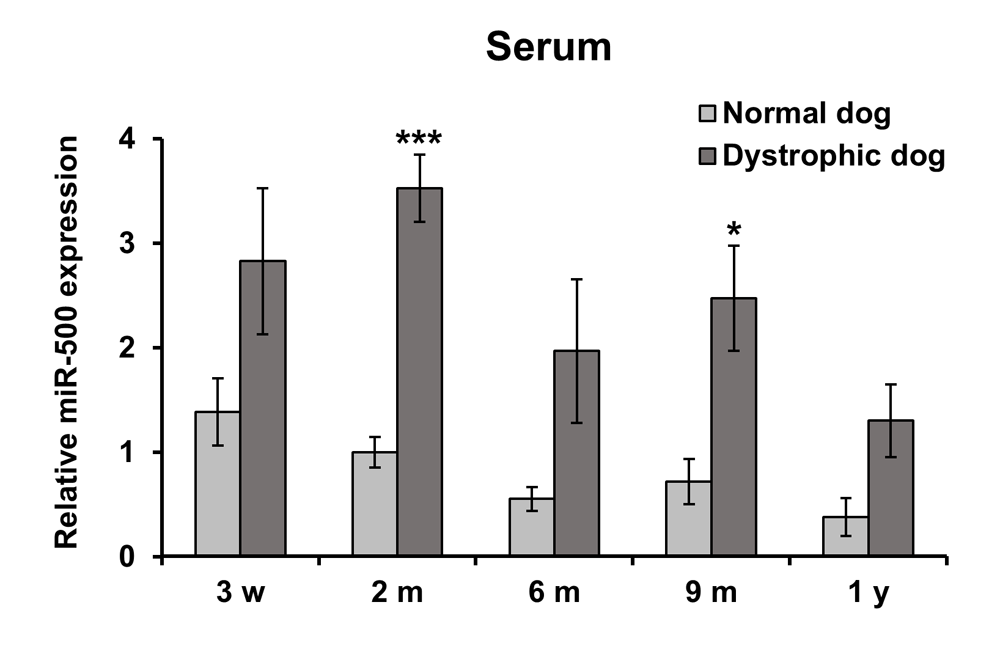

Supplement: S3 Fig — Expression patterns of miR-500 in serum of normal and dystrophic dogs at ages of 3 weeks, 2 months, 6 months, 9 months, and 1 year were analyzed by RT-qPCR (n = 7 each). Data represent means ± SE. Statistical analysis was performed using Student’s t-test with the Holm multiple test; *P < 0.05, ***P < 0.001 compared to normal dogs. (TIF) [file pone.0211597.s003.tif]
