# Supplementary material for: Characterization of a novel microRNA, miR-188, elevated in serum of muscular dystrophy dog model
Source: PLoS One. 2019 Jan 30;14(1):e0211597. doi: 10.1371/journal.pone.0211597 (PMC6353185; doi:10.1371/journal.pone.0211597)
Supplement: S1 Table — List of signal intensities globally normalized on serum miRNA microarray. ND indicates that miRNA was not detected. (PDF) [file pone.0211597.s004.pdf]

S1 Table. miRNA microarray data

miRNA name	miRBase accession	Signal intensity		Fold change
		Normal dog	Dystrophic dog	(Normal dog/Dystrophic dog)
cfa-miR-448	MIMAT0001535	ND	12.464909	-
cfa-miR-429	MIMAT0001539	ND	ND	-
cfa-miR-365	MIMAT0001540	74.937529	120.331237	1.605754001
cfa-miR-449	MIMAT0001544	ND	21.689762	-
cfa-miR-450a	MIMAT0001548	ND	ND	-
cfa-miR-216b	MIMAT0006592	ND	ND	-
cfa-miR-33a	MIMAT0006593	ND	ND	-
cfa-let-7a	MIMAT0006594	34.167443	23.594103	0.690543422
cfa-miR-26a	MIMAT0006595	16.820016	15.302387	0.90977244
cfa-miR-1835	MIMAT0006596	57.954074	48.388357	0.834943148
cfa-miR-32	MIMAT0006597	ND	ND	-
cfa-miR-204	MIMAT0006598	8.997853	19.133138	2.126411489
cfa-miR-31	MIMAT0006599	8.370745	ND	-
cfa-miR-101	MIMAT0006600	12.862527	ND	-
cfa-miR-491	MIMAT0006601	8.301324	ND	-
cfa-miR-150	MIMAT0006602	87.914603	65.351813	0.743355606
cfa-miR-455	MIMAT0006603	ND	8.288250	-
cfa-miR-30a	MIMAT0006604	29.597763	64.884737	2.192217601
cfa-miR-30c	MIMAT0006605	24.622764	21.097237	0.85681839
cfa-miR-206	MIMAT0006606	11.046352	139.265810	12.60740288
cfa-miR-99b	MIMAT0006607	24.168250	31.248382	1.292951786
cfa-let-7e	MIMAT0006608	9.110440	9.887817	1.085328151
cfa-miR-125a	MIMAT0006609	31.163664	36.339019	1.166070171
cfa-let-7f	MIMAT0006610	ND	7.513972	-
cfa-miR-219-5p	MIMAT0006611	ND	ND	-
cfa-miR-23b	MIMAT0006612	153.095393	259.409126	1.694428035
cfa-miR-27b	MIMAT0006613	45.223831	100.958744	2.23242352
cfa-miR-24	MIMAT0006614	468.790965	501.402170	1.069564491
cfa-miR-151	MIMAT0006615	14.167670	14.720651	1.039031189
cfa-miR-30d	MIMAT0006616	79.511048	164.520726	2.069155547
cfa-miR-30b	MIMAT0006617	26.802338	25.677607	0.958036086
cfa-miR-1836	MIMAT0006618	ND	ND	-
cfa-miR-122	MIMAT0006619	8.634454	8.140621	0.94280669
cfa-miR-196b	MIMAT0006620	ND	ND	-
cfa-miR-183	MIMAT0006621	ND	ND	-
cfa-miR-148a	MIMAT0006622	8.463293	7.735119	0.913960913
cfa-miR-129	MIMAT0006623	22.903104	24.052828	1.050199484
cfa-miR-335	MIMAT0006624	ND	ND	-
cfa-miR-29b	MIMAT0006625	9.085666	15.529209	1.709198753
cfa-miR-29a	MIMAT0006626	15.190584	13.094202	0.861994641
cfa-miR-30e	MIMAT0006627	ND	7.898402	-
cfa-miR-135a-3p	MIMAT0006628	11.084701	18.254300	1.646801298
cfa-miR-383	MIMAT0006629	ND	10.134822	-
cfa-miR-1837	MIMAT0006630	151.215636	126.163492	0.834328349
cfa-miR-130a	MIMAT0006631	ND	13.161512	-
cfa-miR-192	MIMAT0006632	30.032277	26.685122	0.888548078
cfa-miR-128	MIMAT0006633	10.423128	63.527362	6.094846192
cfa-miR-7	MIMAT0006634	ND	ND	-
cfa-miR-181c	MIMAT0006635	ND	ND	-
cfa-miR-181d	MIMAT0006636	ND	9.218528	-

cfa-let-7g	MIMAT0006637	16.482336	ND	-
cfa-miR-191	MIMAT0006638	50.108742	37.897083	0.756296835
cfa-miR-425	MIMAT0006639	21.745505	34.059366	1.566271558
cfa-miR-23a	MIMAT0006640	284.078038	275.116201	0.968452904
cfa-miR-27a	MIMAT0006641	79.773952	115.358241	1.446064011
cfa-miR-199	MIMAT0006642	25.483933	12.837527	0.503749833
cfa-miR-708	MIMAT0006643	25.664109	17.540425	0.683461288
cfa-miR-1838	MIMAT0006644	11.389703	11.510556	1.010610724
cfa-miR-139	MIMAT0006645	69.295067	129.658404	1.871105832
cfa-miR-138b	MIMAT0006646	10.925236	13.029157	1.192574421
cfa-miR-15a	MIMAT0006647	17.459977	8.138244	0.466108518
cfa-miR-16	MIMAT0006648	181.560773	62.746178	0.345593252
cfa-miR-17	MIMAT0006649	10.973985	ND	-
cfa-miR-19a	MIMAT0006650	ND	ND	-
cfa-miR-20a	MIMAT0006651	40.890984	14.238455	0.348205243
cfa-miR-19b	MIMAT0006652	31.531840	25.136892	0.797190776
cfa-miR-92a	MIMAT0006653	325.160850	271.662802	0.83547205
cfa-miR-138a	MIMAT0006654	14.944915	26.561292	1.777279563
cfa-miR-499	MIMAT0006655	12.251179	ND	-
cfa-miR-1	MIMAT0006656	ND	20.585239	-
cfa-miR-124	MIMAT0006657	13.994278	18.666371	1.333857381
cfa-miR-320	MIMAT0006658	99.563455	95.586046	0.960051517
cfa-miR-130b	MIMAT0006659	ND	11.220032	-
cfa-miR-185	MIMAT0006660	ND	11.354117	-
cfa-miR-1306	MIMAT0006661	72.495587	109.513490	1.510622847
cfa-miR-196a	MIMAT0006662	ND	7.576846	-
cfa-miR-148b	MIMAT0006663	ND	ND	-
cfa-miR-200c	MIMAT0006664	ND	38.816039	-
cfa-miR-1307	MIMAT0006665	98.397769	112.012792	1.138367192
cfa-miR-107	MIMAT0006666	14.829510	ND	-
cfa-miR-146b	MIMAT0006667	ND	ND	-
cfa-miR-99a	MIMAT0006668	35.052454	44.596681	1.272284132
cfa-let-7c	MIMAT0006669	32.660774	22.339144	0.683974728
cfa-miR-125b	MIMAT0006670	65.698025	72.635851	1.105601744
cfa-miR-155	MIMAT0006671	ND	ND	-
cfa-miR-218	MIMAT0006672	ND	ND	-
cfa-miR-574	MIMAT0006673	105.353952	120.203472	1.140948865
cfa-miR-9	MIMAT0006674	ND	ND	-
cfa-miR-28	MIMAT0006675	11.670776	ND	-
cfa-miR-15b	MIMAT0006676	52.188402	30.038121	0.575570814
cfa-miR-1839	MIMAT0006677	ND	ND	-
cfa-miR-26b	MIMAT0006678	12.430735	ND	-
cfa-miR-1840	MIMAT0006679	19.626780	34.178924	1.741443273
cfa-miR-664	MIMAT0006680	ND	6.871135	-
cfa-miR-194	MIMAT0006681	ND	ND	-
cfa-miR-143	MIMAT0006682	ND	ND	-
cfa-miR-378	MIMAT0006683	57.312689	215.169978	3.75431657
cfa-miR-146a	MIMAT0006684	ND	ND	-
cfa-miR-1271	MIMAT0006685	17.619978	ND	-
cfa-miR-1841	MIMAT0006686	52.098627	49.487111	0.949873612
cfa-miR-103	MIMAT0006687	12.426940	41.381014	3.329943977
cfa-miR-328	MIMAT0006688	126.956328	113.080720	0.890705661
cfa-miR-140	MIMAT0006689	297.010069	134.784654	0.453804999
cfa-miR-34a	MIMAT0006690	11.248228	ND	-

cfa-miR-497	MIMAT0006691	ND	7.286176	-
cfa-miR-195	MIMAT0006692	18.760705	10.487153	0.558995677
cfa-miR-34c	MIMAT0006693	ND	ND	-
cfa-miR-186	MIMAT0006694	17.116893	ND	-
cfa-miR-106b	MIMAT0006695	ND	8.432693	-
cfa-miR-93	MIMAT0006696	74.586990	51.328612	0.688171114
cfa-miR-25	MIMAT0006697	107.291339	74.672650	0.695980223
cfa-miR-197	MIMAT0006698	76.744690	102.279178	1.332719932
cfa-miR-193b	MIMAT0006699	301.268185	279.226411	0.926836702
cfa-miR-590	MIMAT0006700	ND	ND	-
cfa-miR-1842	MIMAT0006701	ND	ND	-
cfa-miR-137	MIMAT0006702	ND	8.469073	-
cfa-miR-92b	MIMAT0006703	207.548053	206.635133	0.995601404
cfa-miR-350	MIMAT0006704	16.790419	19.415508	1.15634446
cfa-miR-29c	MIMAT0006705	13.152798	17.737886	1.34860172
cfa-miR-1843	MIMAT0006706	16.420057	28.031751	1.707165267
cfa-miR-181a	MIMAT0006707	22.498858	17.682881	0.785945713
cfa-miR-181b	MIMAT0006708	14.098388	ND	-
cfa-miR-342	MIMAT0006709	30.110085	36.235054	1.203419187
cfa-miR-345	MIMAT0006710	63.528232	77.274791	1.21638504
cfa-miR-493	MIMAT0006711	ND	7.505490	-
cfa-miR-433	MIMAT0006712	ND	21.558390	-
cfa-miR-127	MIMAT0006713	22.347117	80.909486	3.620578261
cfa-miR-136	MIMAT0006714	ND	ND	-
cfa-miR-379	MIMAT0006715	15.510318	15.902196	1.025265633
cfa-miR-411	MIMAT0006716	ND	19.921779	-
cfa-miR-380	MIMAT0006717	ND	23.028985	-
cfa-miR-323	MIMAT0006718	ND	ND	-
cfa-miR-329a	MIMAT0006719	ND	ND	-
cfa-miR-543	MIMAT0006720	ND	9.053677	-
cfa-miR-495	MIMAT0006721	ND	ND	-
cfa-miR-376a	MIMAT0006722	ND	ND	-
cfa-miR-487b	MIMAT0006723	23.109312	22.481426	0.972829741
cfa-miR-382	MIMAT0006724	ND	13.899522	-
cfa-miR-485	MIMAT0006725	13.356285	22.011749	1.648044273
cfa-miR-409	MIMAT0006726	17.666408	44.705020	2.530509881
cfa-miR-369	MIMAT0006727	ND	ND	-
cfa-miR-410	MIMAT0006728	ND	14.316931	-
cfa-miR-219-3p	MIMAT0006729	ND	ND	-
cfa-miR-126	MIMAT0006730	ND	ND	-
cfa-miR-212	MIMAT0006731	ND	14.027666	-
cfa-miR-132	MIMAT0006732	ND	8.365927	-
cfa-miR-22	MIMAT0006733	37.353724	170.576996	4.566532536
cfa-miR-144	MIMAT0006734	ND	ND	-
cfa-miR-193a	MIMAT0006735	135.565145	178.715703	1.318301271
cfa-miR-142	MIMAT0006736	12.798733	28.682424	2.241036203
cfa-miR-10a	MIMAT0006737	9.972503	9.730526	0.97573558
cfa-miR-152	MIMAT0006738	ND	ND	-
cfa-miR-338	MIMAT0006739	ND	ND	-
cfa-miR-1844	MIMAT0006740	243.939430	277.234364	1.136488529
cfa-miR-21	MIMAT0006741	22.006850	8.870090	0.403060411
cfa-miR-423a	MIMAT0006742	136.126805	147.017097	1.080001084
cfa-miR-652	MIMAT0006743	11.266396	8.656544	0.768350766
cfa-miR-224	MIMAT0006744	ND	ND	-

cfa-miR-424	MIMAT0006745	11.541668	7.766478	0.672907763
cfa-miR-503	MIMAT0006746	65.932093	123.415987	1.871865148
cfa-miR-542	MIMAT0006747	ND	15.138991	-
cfa-miR-450b	MIMAT0006748	ND	ND	-
cfa-miR-106a	MIMAT0006749	43.594622	20.014034	0.459094106
cfa-miR-363	MIMAT0006750	ND	ND	-
cfa-miR-361	MIMAT0006751	14.965224	9.428620	0.630035341
cfa-miR-384	MIMAT0006752	ND	ND	-
cfa-miR-374a	MIMAT0006753	ND	ND	-
cfa-miR-374b	MIMAT0006754	ND	12.917959	-
cfa-miR-421	MIMAT0006755	ND	ND	-
cfa-miR-98	MIMAT0006756	ND	ND	-
cfa-miR-221	MIMAT0006757	126.460116	70.239962	0.555431738
cfa-miR-532	MIMAT0006758	14.477272	16.705795	1.153932523
cfa-miR-500	MIMAT0006759	11.155412	25.806990	2.313405368
cfa-miR-660	MIMAT0006760	10.296524	10.451255	1.015027499
cfa-miR-502	MIMAT0006761	16.429384	28.684843	1.745947566
cfa-miR-676	MIMAT0006762	ND	ND	-
cfa-let-7j	MIMAT0006763	ND	ND	-
cfa-miR-371	MIMAT0007747	12.942259	16.617484	1.283970905
cfa-miR-20b	MIMAT0009830	30.206383	16.557192	0.548135538
cfa-miR-18b	MIMAT0009831	ND	ND	-
cfa-miR-18a	MIMAT0009832	14.678657	12.526707	0.853395988
cfa-miR-133c	MIMAT0009833	13.762044	2096.678914	152.3522897
cfa-miR-133a	MIMAT0009834	15.064242	1934.238540	128.3993274
cfa-miR-133b	MIMAT0009835	27.836138	2425.674410	87.14119789
cfa-let-7b	MIMAT0009836	39.010696	18.154606	0.465375086
cfa-miR-10b	MIMAT0009837	25.519196	52.692412	2.064814738
cfa-miR-34b	MIMAT0009838	ND	ND	-
cfa-miR-135b	MIMAT0009839	ND	ND	-
cfa-miR-153	MIMAT0009840	ND	12.636235	-
cfa-miR-182	MIMAT0009841	ND	ND	-
cfa-miR-184	MIMAT0009842	15.528486	12.069611	0.777256134
cfa-miR-187	MIMAT0009843	37.079339	24.863108	0.670538059
cfa-miR-202	MIMAT0009844	ND	9.516655	-
cfa-miR-205	MIMAT0009845	46.828847	39.353497	0.840368694
cfa-miR-210	MIMAT0009846	17.905784	25.383906	1.417637228
cfa-miR-214	MIMAT0009847	38.838110	45.082873	1.16078957
cfa-miR-215	MIMAT0009848	ND	ND	-
cfa-miR-216a	MIMAT0009849	ND	ND	-
cfa-miR-217	MIMAT0009850	ND	15.322897	-
cfa-miR-222	MIMAT0009851	14.625710	ND	-
cfa-miR-223	MIMAT0009852	205.714017	112.889960	0.548771356
cfa-miR-301a	MIMAT0009853	ND	ND	-
cfa-miR-301b	MIMAT0009854	ND	ND	-
cfa-miR-302a	MIMAT0009855	ND	ND	-
cfa-miR-302b	MIMAT0009856	ND	ND	-
cfa-miR-302c	MIMAT0009857	67.970548	50.077802	0.736757367
cfa-miR-302d	MIMAT0009858	ND	18.371164	-
cfa-miR-367	MIMAT0009859	ND	ND	-
cfa-miR-489	MIMAT0009860	133.519905	127.146304	0.952264788
cfa-miR-96	MIMAT0009861	ND	ND	-
cfa-miR-33b	MIMAT0009862	ND	ND	-
cfa-miR-145	MIMAT0009863	73.593589	37.218718	0.505733156

cfa-miR-200b	MIMAT0009864	ND	ND	-
cfa-miR-200a	MIMAT0009865	ND	12.303726	-
cfa-miR-203	MIMAT0009866	23.632354	13.465407	0.569786954
cfa-miR-211	MIMAT0009867	25.000000	30.370087	1.21480348
cfa-miR-208a	MIMAT0009868	ND	ND	-
cfa-miR-208b	MIMAT0009869	ND	ND	-
cfa-miR-451	MIMAT0009870	1275.829643	250.036134	0.195979248
cfa-miR-375	MIMAT0009871	ND	ND	-
cfa-miR-190a	MIMAT0009872	8.661286	ND	-
cfa-miR-190b	MIMAT0009873	ND	9.731336	-
cfa-miR-147	MIMAT0009874	ND	ND	-
cfa-miR-490	MIMAT0009875	ND	ND	-
cfa-miR-141	MIMAT0009876	ND	7.042942	-
cfa-miR-514	MIMAT0009877	ND	7.173902	-
cfa-miR-95	MIMAT0009878	ND	ND	-
cfa-miR-105a	MIMAT0009879	9.374060	42.414456	4.524662313
cfa-miR-188	MIMAT0009880	30.728352	76.291342	2.48276712
cfa-miR-134	MIMAT0009883	15.650899	24.041539	1.536112335
cfa-miR-149	MIMAT0009884	44.182129	41.847617	0.947161623
cfa-miR-299	MIMAT0009885	18.931733	37.604734	1.986333422
cfa-miR-362	MIMAT0009886	8.491376	16.722482	1.969348902
cfa-miR-376b	MIMAT0009887	ND	ND	-
cfa-miR-376c	MIMAT0009888	ND	ND	-
cfa-miR-370	MIMAT0009889	2975.724503	1501.776269	0.504675842
cfa-miR-377	MIMAT0009890	12.687257	7.680329	0.605357722
cfa-miR-381	MIMAT0009891	ND	38.241087	-
cfa-miR-340	MIMAT0009892	ND	ND	-
cfa-miR-330	MIMAT0009893	62.072281	96.626076	1.556670295
cfa-miR-326	MIMAT0009894	59.762980	82.418701	1.379092893
cfa-miR-331	MIMAT0009895	28.697888	37.323984	1.300582956
cfa-miR-324	MIMAT0009896	ND	12.615889	-
cfa-miR-325	MIMAT0009897	ND	ND	-
cfa-miR-346	MIMAT0009898	81.779956	99.044035	1.211104039
cfa-miR-329b	MIMAT0009899	ND	ND	-
cfa-miR-452	MIMAT0009900	ND	13.971033	-
cfa-miR-483	MIMAT0009901	91.810618	118.979545	1.295923583
cfa-miR-487a	MIMAT0009902	14.201917	ND	-
cfa-miR-488	MIMAT0009903	11.073540	17.604015	1.589736886
cfa-miR-432	MIMAT0009904	ND	9.038678	-
cfa-miR-494	MIMAT0009905	69.168323	54.890188	0.793574076
cfa-miR-496	MIMAT0009906	ND	ND	-
cfa-miR-504	MIMAT0009907	ND	ND	-
cfa-miR-505	MIMAT0009908	51.951038	34.265616	0.659575195
cfa-miR-539	MIMAT0009909	15.571614	21.216366	1.362502692
cfa-miR-544	MIMAT0009910	ND	ND	-
cfa-miR-545	MIMAT0009911	ND	ND	-
cfa-miR-551a	MIMAT0009912	22.181360	34.998977	1.577855325
cfa-miR-551b	MIMAT0009913	20.104376	21.475469	1.068198734
cfa-miR-568	MIMAT0009914	ND	ND	-
cfa-miR-578	MIMAT0009915	15.733844	ND	-
cfa-miR-582	MIMAT0009916	ND	ND	-
cfa-miR-589	MIMAT0009917	ND	ND	-
cfa-miR-592	MIMAT0009918	ND	ND	-
cfa-miR-599	MIMAT0009919	ND	ND	-

cfa-miR-615	MIMAT0009920	507.107955	526.170405	1.037590517
cfa-miR-628	MIMAT0009921	ND	ND	-
cfa-miR-631	MIMAT0009922	29.182574	30.980324	1.061603545
cfa-miR-632	MIMAT0009923	ND	12.902020	-
cfa-miR-653	MIMAT0009924	ND	ND	-
cfa-miR-758	MIMAT0009925	ND	ND	-
cfa-miR-671	MIMAT0009926	39.725663	40.387862	1.0166693
cfa-miR-454	MIMAT0009927	ND	7.247687	-
cfa-miR-802	MIMAT0009928	ND	7.871641	-
cfa-miR-300	MIMAT0009929	ND	ND	-
cfa-miR-874	MIMAT0009930	95.646283	127.844422	1.33663764
cfa-miR-875	MIMAT0009931	ND	ND	-
cfa-miR-876	MIMAT0009932	ND	ND	-
cfa-miR-885	MIMAT0009933	52.284744	82.013045	1.568584614
cfa-miR-665	MIMAT0009934	31.880993	35.266070	1.106178531
cfa-miR-207	MIMAT0009935	52.150947	57.371113	1.100097243
cfa-miR-761	MIMAT0009936	24.922810	14.878424	0.596980196
cfa-miR-764	MIMAT0009937	15.396792	26.122503	1.696619854
cfa-miR-759	MIMAT0009938	ND	9.150194	-
cfa-miR-718	MIMAT0009939	408.268091	400.326827	0.980548899
cfa-miR-872	MIMAT0009940	ND	25.822265	-
cfa-miR-135a-5p	MIMAT0010196	ND	18.764610	-
cfa-miR-105b	MIMAT0010198	ND	ND	-
cfa-miR-1199-5p	MIMAT0031123	10.215942	19.974080	1.955187295
cfa-miR-1199-3p	MIMAT0031124	9.581693	12.074897	1.260204955
