# Supplementary material for: Characterization of a novel microRNA, miR-188, elevated in serum of muscular dystrophy dog model
Source: PLoS One. 2019 Jan 30;14(1):e0211597. doi: 10.1371/journal.pone.0211597 (PMC6353185; doi:10.1371/journal.pone.0211597)
Supplement: S2 Table — (PDF) [file pone.0211597.s005.pdf]

S2 Table. List of primer sets for 18S rRNA and mRNAs

Target	NCBI GeneBank Accession #	Primer Sequence		Reference
18S rRNA	NR_003278	Forward	TTGATTAAGTCCCTGCCCTTTGT	-
		Reverse	CCGATCCGAGGGCCTCACTA	
MyoD	NM_010866	Forward	GCTCTGATGGCATGATGGAT	Roberts TC et al., 2013
		Reverse	GACACAGCCGCACTCTT	
Myf5	NM_008656	Forward	CACCTCCAACTGCTCTGAC	Roberts TC et al., 2013
		Reverse	ACATGCATTTGATACATCAGGAC	
Myog	NM_031189	Forward	CGATCTCCGCTACAGAGG	Roberts TC et al., 2013
		Reverse	CGCGAGCAAATGATCTCCT	
MRF4	NM_008657	Forward	CATCAGCTACATTGAGCGTCTACAGG	Yuasa K et al., 2015
		Reverse	CTGGAATGATCCGAAACACTTGG	
MEF2C	NM_001170537	Forward	TGGCAGCAAGAACACGATGC	Yuasa K et al., 2015
		Reverse	AGGAGTTGCTACGGAAACCAC	
Ckm	NM_007710	Forward	CAGCAGCTCATTGATGACCACTTCCTG	Yuasa K et al., 2015
		Reverse	GCAGAAGCGGCGGAAAACCTCCTTC	
Myh1	NM_030679	Forward	GTCCAAAGCCAACAGTGAAG	Yuasa K et al., 2015
		Reverse	CTTCTGTTTCCATTCTGCCA	
Myh2	NM_001039545	Forward	AGGCGGCTGAGGAGCACGTA	Brown DM et al., 2012
		Reverse	GCGGCACAAGCAGCGTTGG	
Myh4	NM_010855	Forward	CAATCAGGAACCTTCGGAACAC	Brown DM et al., 2012
		Reverse	GTCCTGGCCTCTGAGAGCAT	
Mouse MITR	NM_024124	Forward	GGAGCTAGACGCCAGGTTTA	-
		Reverse	GCAGGTGGGCAAAAGGAATG	
Dog MITR	XM_022427451	Forward	ATCCCAAAGCCTGTCACACC	-
		Reverse	TCCAGCAGTCACTGATCTCAAC	
SUMO2	NM_133354	Forward	CAGCCAATCAACGAAACAGA	La Salle S et al., 2008
		Reverse	ATGTGGTGGGACCAAATTGT	
UBE2I	NM_011665	Forward	TCTCCCTGCCTGTTAGCTGT	La Salle S et al., 2008
		Reverse	GAATGCAGGTCAGGAGGTGT	

References

Roberts TC, Godfrey C, McClorey G, Vader P, Briggs D, Gardiner C, et al. Extracellular microRNAs are dynamic non-vesicular biomarkers of muscle turnover. Nucleic Acids Res. 2013;41(20):9500-13. doi: 10.1093/nar/gkt724.

Yuasa K, Aoki N, Hijikata T. JAZF1 promotes proliferation of C2C12 cells, but retards their myogenic differentiation through transcriptional repression of MEF2C and MRF4-Implications for the role of Jazf1 variants in oncogenesis and type 2 diabetes. Exp Cell Res. 2015;336(2):287-97. doi: 10.1016/j.yexcr.2015.06.009.

Brown DM, Parr T, Brameld JM. Myosin heavy chain mRNA isoforms are expressed in two distinct cohorts during C2C12 myogenesis. J Muscle Res Cell Motil. 2012;32(6):383-90. doi: 10.1007/s10974-011-9267-4.

La Salle S, Sun F, Zhang XD, Matunis MJ, Handel MA. Developmental control of sumoylation pathway proteins in mouse male germ cells. Dev Biol. 2008;321(1):227-37. doi: 10.1016/j.ydbio.2008.06.020.
