# Supplementary material for: Characterization of a novel microRNA, miR-188, elevated in serum of muscular dystrophy dog model
Source: PLoS One. 2019 Jan 30;14(1):e0211597. doi: 10.1371/journal.pone.0211597 (PMC6353185; doi:10.1371/journal.pone.0211597)
Supplement: S3 Table — (PDF) [file pone.0211597.s006.pdf]

S3 Table. List of primer sets for snoRNAs and miRNAs

Target	Primer name	Catalog number
cfa-miR-10b	Cf_miR-10b_1 miScript Primer Assay	MS00029393
cfa-miR-103	Cf_miR-103_1 miScript Primer Assay	MS00029358
cfa-miR-105a	Cf_miR-105a_1 miScript Primer Assay	MS00029365
cfa-miR-142	Cf_miR-142_1 miScript Primer Assay	MS00029589
cfa-miR-188	Cf_miR-188_1 miScript Primer Assay	MS00029841
mmu-miR-188-5p	Mm_miR-188_1 miScript Primer Assay	MS00001757
cfa-miR-204	Cf_miR-204_1 miScript Primer Assay	MS00029995
cfa-miR-500	Cf_miR-500_1 miScript Primer Assay	MS00030856
sno202	Hs_SNORD68_11 miScript Primer Assay	MS00033712
sno234	Custom miScript Primer Assay	MSC0075968
